# Supplementary material for: Microstructure and Tensile Properties of Melt-Spun Filaments of Polybutene-1 and Butene-1/Ethylene Copolymer
Source: Polymers (Basel). 2023 Sep 11;15(18):3729. doi: 10.3390/polym15183729 (PMC10536619; doi:10.3390/polym15183729)
Supplement: Supplementary file 1 [file polymers-15-03729-s001.zip › polymers-2569238-supplementary.pdf]

Supporting Information

Figure S1 showed the SEM micrographs of the cross-sections of PB-1 filaments, and the FE-SEM micrographs of the surface of PB-1 films as shown in Figure S2. It is observed in Figure S1 that the cross-sections of both filaments are circular, and no obvious defects appear. It is seen in Figure S2 that the surface of the PB8220M film presents many small sheet-like structures attached, while the surface of the PB0110M film is smooth and flat. It may be because of the ethylene comonomer does not participate in the crystallization of the polybutene-1 chain segments and is excluded into the amorphous region.

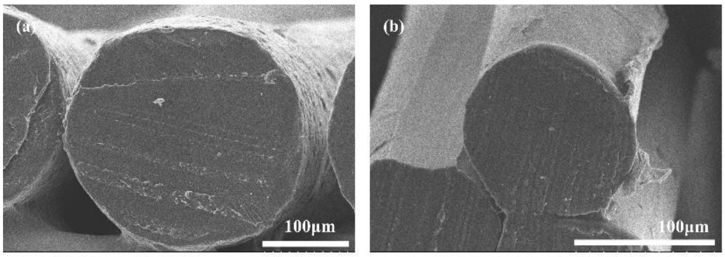

Figure S1. SEM micrographs of the cross-sections of PB0110M filaments (a) and PB8220M filaments (b).

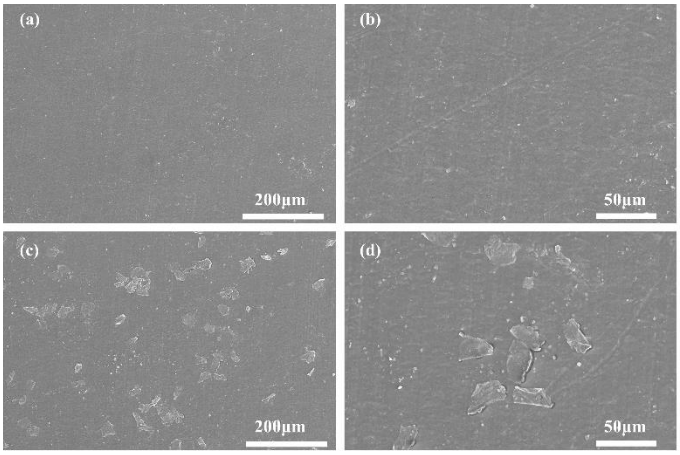

Figure S2. FE-SEM micrographs of the surface of PB0110M films (a, b) and PB8220M films (c, d).
